# Sandwich-Type Electrochemical Aptasensor with Supramolecular Architecture for Prostate-Specific Antigen

**DOI:** 10.3390/molecules29194714

**Published:** 2024-10-05

**Authors:** Anabel Villalonga, Raúl Díaz, Irene Ojeda, Alfredo Sánchez, Beatriz Mayol, Paloma Martínez-Ruiz, Reynaldo Villalonga, Diana Vilela

**Affiliations:** 1 Nanosensors and Nanomachines Group, Department of Analytical Chemistry, Faculty of Chemistry, Complutense University of Madrid, 28040 Madrid, Spainbeamayol@ucm.es (B.M.); 2 Faculty of Pharmaceutical Sciences, Complutense University of Madrid, 28040 Madrid, Spain; 3 Department of Organic Chemistry I, Faculty of Chemistry, Complutense University of Madrid, 28040 Madrid, Spain

**Keywords:** aptamer, electrochemical biosensor, prostate-specific antigen, gold nanoparticles, amperometry, β-cyclodextrin, supramolecular

## Abstract

A novel sandwich-type electrochemical aptasensor based on supramolecularly immobilized affinity bioreceptor was prepared via host–guest interactions. This method utilizes an adamantane-modified, target-responsive hairpin DNA aptamer as a capture molecular receptor, along with a perthiolated β-cyclodextrin (CD) covalently attached to a gold-modified electrode surface as the transduction element. The proposed sensing strategy employed an enzyme-modified aptamer as the signalling element to develop a sandwich-type aptasensor for detecting prostate-specific antigen (PSA). To achieve this, screen-printed carbon electrodes (SPCEs) with electrodeposited reduced graphene oxide (RGO) and gold nanoferns (AuNFs) were modified with the CD derivative to subsequently anchor the adamantane-modified anti-PSA aptamer via supramolecular associations. The sensing mechanism involves the affinity recognition of PSA molecules on the aptamer-enriched electrode surface, followed by the binding of an anti-PSA aptamer–horseradish peroxidase complex as a labelling element. This sandwich-type arrangement produces an analytical signal upon the addition of H_2_O_2_ and hydroquinone as enzyme substrates. The aptasensor successfully detected the biomarker within a concentration range of 0.5 ng/mL to 50 ng/mL, exhibiting high selectivity and a detection limit of 0.11 ng/mL in PBS.

## 1. Introduction

The design and development of surface-based biosensors is challenging. Non-specific binding (NSB) is a complex persistent problem found in nature that negatively affects biosensor performance in terms of sensitivity, specificity, and reproducibility [1]. Among the surface-based biosensors, electrochemical biosensors have aroused great interest for the design and development of point of care diagnosis devices since they have fast responses as a consequence of short detection times, low cost, great potential for miniaturization and real sample analysis, high portability, and ease of operation, apart from high sensitivity and specificity, accuracy, and low detection limits [2]. In this sense, electrochemical biosensors, which are prepared via the immobilization of a capture probe onto an electrode surface to enhance probe–surface interactions and avoid undesired phenomena such as fouling or NSB, have resulted in highly complex and heterogeneous electrode surfaces [3]. These heterogeneous surfaces can provoke other unexpected probe–surface interactions that are usually ignored. The most common approaches dedicated to reducing NSB are proper surface engineering, the use of blocking agents, and sample pre-treatment [4]. However, despite the use of those approaches, NSB is still prevalent. Thus, it is important to design and develop new methods to modify the electrode surface in a controllable manner in order to remove weakly non-specific adsorptions.

Over the last two decades, the use of nanomaterials has had a great impact on biosensing. Nanomaterials are commonly used as electrode surface modifiers because they provide a large surface area that leads to increased loading capacity, fouling resistance, and mass transport of reactants, which results in an improvement in the analytical performance of electrochemical biosensors [5]. The most widely used nanomaterials for the development of electrochemical biosensors are carbon-based nanomaterials, metallic nanoparticles, mesoporous silica nanoparticles, and hybrid nanocomposites, among others [6]. Regarding carbon-based nanomaterials, reduced graphene oxide (rGO) is widely employed in sensing applications due to its excellent electrical conductivity, large theoretical specific surface area, hydrophilic behaviour, and strong mechanical resistance [7]. Graphene oxide (GO) and rGO contain hydroxyl, epoxy, carbonyl, and carboxyl moieties in different quantities that influence the electrochemical performance in terms of electron transfer rate or adsorption/desorption of molecules and offer functionalization sites with the potential for biomolecule anchoring [8]. Therefore, rGO has been used as a promising electrode material in several electrochemical sensing and biosensing applications such as glucose sensors [9,10], peroxide, uric acid, ascorbic acid, metal ions, NADH, pesticides, etc. [11]. Novel metal nanoparticles, particularly gold nanoparticles (AuNPs), are also integrated as electrode modifiers for the design of electrochemical (bio-)sensors because of their unique catalytic, electric, and surface-related properties. Thus, AuNPs can act as signal amplifiers, electrocatalysts. and support for the immobilization of biomolecules [12,13,14]. As a promising strategy, AuNPs have been electrodeposited onto rGO to produce huge surface ratios and unique binding sites on the electrode surface [15,16], while also causing the separation of re-stacked rGO sheets [17,18].

The assembly of the capture biomolecular elements on the electrode surface can be carried out through covalent or non-covalent linkages. Regarding dynamic non-covalent interactions, supramolecular materials are promising tools to advance the capabilities of the currently available biosensors [19]. This approach was reported as an efficient and reversible method for the immobilization of enzymes, resulting in the construction of suitable catalytic electrochemical biosensors [20]. This strategy involved the complementary interaction of adamantane derivatives with β-cyclodextrin, which is well known as the most relevant molecular receptor in supramolecular chemistry [21], for the immobilization of bioreceptors. Currently, despite the advantages that supramolecular chemistry offers, there are a few works that have developed supramolecular based-affinity electrochemical biosensors. For example, Ortiz et al. reported the detection of antigliadin autoantibodies in celiac patient samples by a cyclodextrin-based supramolecular electrochemical immunosensor prepared on gold electrodes [22], while Gao and coworkers, through the design of an electrochemical immunoassay, detected the carcinoembryonic antigen (CEA) using the host–guest interaction between β-cyclodextrin and adamantine-modified antibodies on a glassy carbon electrode (GCE) previously functionalized with graphene [23]. Considering electrochemical aptasensors, Xue et al. designed an electrochemical thrombin aptasensor using cyclodextrin-functionalized graphene–gold nanoparticle hybrids [24]. Recently, Lui et al. developed specific host–guest interactions between cucurbit uril and a ferrocene-based electrochemical aptasensor for the determination of exosomes [25].

**Scheme 1:**
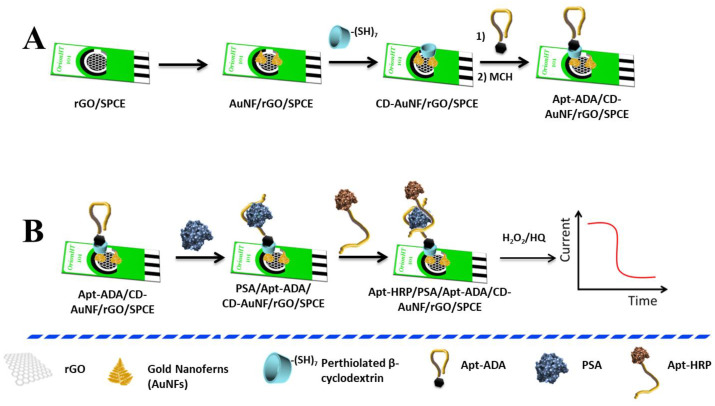
Schematic representation of the processes involved in the assembly (**A**) and method of use (**B**) of the aptasensor for PSA.

In this work, we have proposed the preparation of an electrochemical aptasensor through the immobilization of biomolecules via supramolecular host–guest interactions to avoid typical non-specific adsorption. This approach relies on the use of an adamantane-modified target-responsive hairpin DNA aptamer as an affinity bioreceptor and a perthiolated β-cyclodextin (CD) covalently attached to a gold-modified electrode surface. As a proof of concept, we have developed a sandwich-type amperometric aptasensor for sensing prostate-specific antigens (PSAs) using the proposed strategy (Scheme 1). To this end, the assembly of this sensor was carried out on screen-printed carbon electrodes (SPCE) with previously electrodeposited rGO and fern-like gold nanoparticles (AuNFs), that were modified with CD for the subsequent anchorage of an adamantane-modified anti-PSA aptamer (Apt–ADA) through supramolecular interactions. The sensing mechanism is based on the recognition of the PSA by the capture aptamer on the electrode surface, followed by the recognition of an anti-PSA aptamer–horseradish peroxidase (Apt–HRP) complex as a labelling element thus provoking the analytical signal when the enzyme substrates (H_2_O_2_/hydroquinone) are added.

## 2. Results and Discussion

Scheme 1A displays the experimental process involved in the assembly and working mechanism for the proposed PSA aptasensor. To assemble the electroanalytical device (Scheme 1A), previously activated SPCE was first coated with rGO through the electrodeposition of GO. AuNFs were then electrodeposited on rGO/SPCE by the amperometric reduction of HAuCl_4_ [26]. AuNFs/rGO/SPCE was further functionalized with heptakis(6-deoxy-6-thio)-β-cyclodextrin (CD) through an Au–S bond, and subsequently the Apt–ADA complex was attached to the electrode surface through supramolecular interactions. Finally, to prevent non-specific adsorption on the electrode surface, mercarptohexanol (MCH) was used as blocking agent.

The sensing mechanism proposed in this work for the specific recognition of PSA is displayed in Scheme 1B. As observed, the capture aptamer attached on the electrode surface through supramolecular interactions first recognises PSA, and then an enzyme modified recognition aptamer is added as the labelling element to assemble a sandwich-type architecture. In the end, after the addition of H_2_O_2_/hydroquinone, the amperometric analytical signal is recorded by monitoring the reduction in the enzymatically produced benzoquinone on the sensor surface.

**Figure 1:**
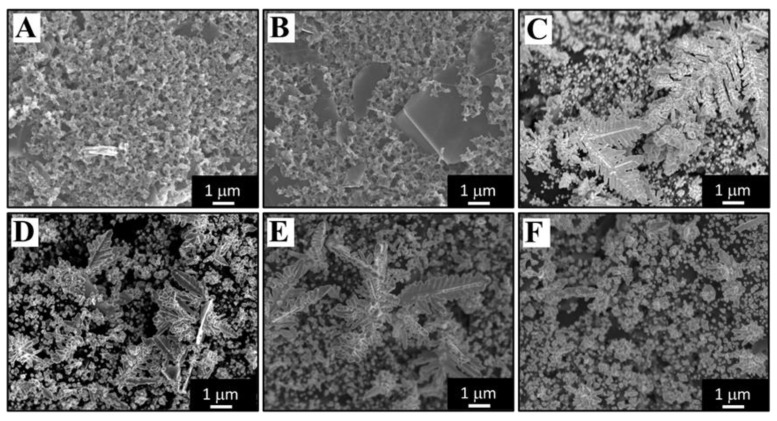
Representative FE-SEM images of bare SPCE (**A**), rGO/SPCE (**B**), AuNFs/rGO/SPCE (**C**), CD–AuNFs/rGO/SPCE (**D**), Apt–ADA/CD–AuNFs/rGO/SPCE (**E**), MCH/Apt–ADA/CD–AuNFs/rGO/SPCE (**F**).

In order to characterize the assembling steps involved in the preparation of the aptasensor for PSA, field emission scanning electron microscopy (FE-SEM) was employed and the representative images are shown in Figure 1 and Figure S1. Figure 1A displays the three-dimensional arrangement of carbon nanoparticles on SPCE, demonstrating a highly roughened surface for this electrode. Such nanoparticles were further coated with rGO nanosheets after the electrochemical deposition of GO (Figure 1B). Subsequently, the electrochemical reduction of HAuCl_4_ led to electrodeposition of Au nanoparticles with a flower-like morphology that have been defined as gold nanoferns (AuNFs) (Figure 1C). Sequential functionalization with the perthiolated CD derivative (Figure 1D), capture Apt–ADA (Figure 1E), and MCH (Figure 1F) did not cause significant differences in the surface morphology of the working electrode. However, the non-conductive properties of these organic molecules resulted in a smoothing effect, resulting a low resolution of the FE-SEM images.

To achieve the accurate and sensitive detection of the PSA, different experimental conditions to prepare the PSA aptasensor were examined and optimized such as the concentrations of Apt–ADA and Apt–HRP and the incubation times of Apt–ADA, PSA, and Apt–HRP, all in the presence of 20 ng/mL PSA (Table 1 and Figure S2 in Supporting Information). The optimal values are reported in Table 1. As shown in Figure S2, the current increased as the concentration of the Apt–ADA complex used was higher (Figure S2A). However, the tendency changed at concentrations higher than 30 μM, so 30 μM was taken as the optimum concentration. A different behaviour was observed when the Apt–HRP concentration was increased, reaching a maximum response when the Apt–HRP concentration was 5 μM, which was then taken as the optimal concentration for sensor construction (Figure S2D). For the incubation time of Apt–ADA and Apt–HRP, a similar trend was observed, obtaining maximum amperometric responses at 60 and 30 min, respectively (Figure S2B,E). And finally, for the incubation time of the target analyte, the maximum signal was reached after 60 min, which was then selected as the optimal incubation time for PSA.

To further demonstrate the proper performance of the PSA sensor under the optimal conditions in the presence and absence of hydroquinone as a redox mediator, two additional experiments were carried out (Figure S2F). On the one hand, the relative amperometric responses of Apt–HRP/PSA/Apt–ADA/CD–AuNF/rGO/SPCE (sample) and Apt–HRP/Apt–ADA/CD–AuNF/rGO/SPCE (control) were measured in the presence of hydroquinone solution and after the addition of 5 µL of a freshly prepared 50 mM H_2_O_2_ solution in 0.1 M sodium phosphate buffer, pH 7.4. On the other hand, the same assays were carried out but in the absence of hydroquinone. Figure S2F displays how the hydroquinone presence is required to obtain a sensible amperometric signal for the PSA detection. Furthermore, Figure S2F (left) demonstrated the proper performance of the proposed PSA aptasensor both in the presence and absence of the target analyte in the sample. This suggests minimal non-specific adsorption on the modified electrode surface, resulting in amperometric signals that are 25% for the control compared to the sample.

**Figure 2:**
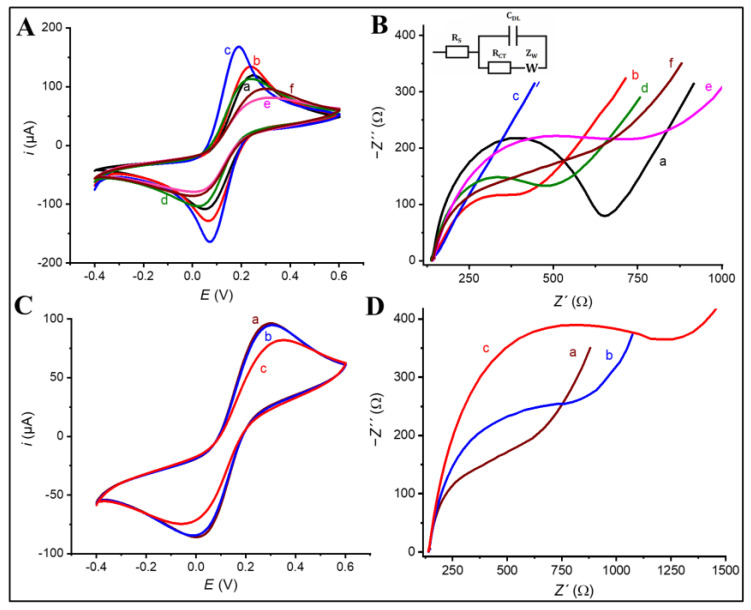
Cyclic voltammograms (**A**) and Nyquist plots (**B**) of SPCE before (a) and after sequential modification with rGO (b), AuNFs (c), CD (d), Apt–ADA (e), and MCH (f). Cyclic voltammograms (**C**) and Nyquist plots (**D**) of the sensor (a) and further incubation with PSA (b) and Apt–HRP (c). Measured in 0.1 M KCl solution containing 5 mM K_3_[Fe(CN)_6_]/K_4_[Fe(CN)_6_] (1:1), (**A**,**C**) scan rate  =  50 mV·s^−1^. Conditions for EIS (**B**,**D**): frequency range of 0.01 to 106 Hz at a fixed potential.

After proper optimization, cyclic voltammetry (CV) and electrochemical impedance spectroscopy (EIS) techniques were employed to characterize the stages involved in the aptasensor assembly as well as its further use (Figure 2). The CVs and EIS of each assembly stage of the aptasensor were driven in a saline solution containing [Fe(CN)_6_]^3−^/[Fe(CN)_6_]^4−^ redox couple. In the CV experiments, a conventional pair of redox peaks was observed for all the electrodes, with quasi-reversible and diffusion-limited cyclic voltammetric behaviours (Figure 2A). In comparison with SPCE (curve a), a notable increase in the current intensity of the redox peaks were observed for rGO/SPCE (curve b) and AuNFs/rGO/SPCE (curve c), which indicates the enhancement of the electron transfer in the presence of these nanomaterials due to increments in the electroactive surface area. In this sense, it was further demonstrated that the electroactive surface area of the electrode, calculated by applying the Randles–Sevcik equation, varied from 12.2 mm^2^ to 13.8 mm^2^ and 17.7 mm^2^ upon the sequential electrodeposition of rGO and AuNFs, repectively. However, this behaviour changed after sequential functionalization of the electrodes with organic molecules. Therefore, the redox peak current intensity decreased, and the electroactive surface reached 11.8 mm^2^, 7.8 mm^2^, and 9.5 mm^2^ values after modifications with CD (curve d), Apt–ADA (curve e), and MCH (curve f), respectively. These results suggest a high coverage of the electrode surface with the organic coating agents and thus the successful preparation of the PSA aptasensor. On the other hand, EIS studies have revealed the charge transfer resistance of the electrode during each assembly stage of the aptasensor preparation by the measure of the semicircle diameter shown in the Nyquist plot (Figure 2B). As illustrated in Figure 2B, the semicircle diameter observed at high frequencies in the Nyquist corresponding to the bare electrode was progressively reduced from 498 Ω to 471 Ω and 25.6 Ω (Figure 2(Ba), Figure 2(Bb), and Figure 2(Bc), respectively) after the electrodeposition of rGO and AuNFs, respectively. This reduced electron transfer resistance agrees with the results obtained using CV as these nanomaterials can improve the electroconductive properties of the electrode surface. On the contrary, surface modification with the non-conductive molecules such as CD, Apt–ADA, and MCH produced a certain increase in the electron transfer resistance, reaching values of 373 Ω, 681 Ω, and 798 Ω, respectively (Figure 2(Bd), Figure 2(Be), and Figure 2(Bf), respectively). In addition, the heterogenous transfer constant (k_0_) was calculated using Equation (1) and Equation (2) based on Banks’ methodology [27,28], using Nicholson and Perone data [29,30,31] on the relationship between Ψ and n·ΔE (Figure S4) in the range from 98 to 290 mV (R^2^ = 0.9947) according to our experimental values of ΔE (Equation (2)).

(1) $$k_{0}=\Psi \sqrt{\frac{D_{0}\pi \nu F}{RT}{\left(\frac{D_{Red}}{D_{Ox}}\right)}^{\alpha }}$$

(2) $$\Psi =25334\times {\left(n∆E\right)}^{-2.32}$$

where D_0_ is the average diffusion coefficient for the ferricyanide/ferrocyanide redox reaction, ν is the scan rate (V⋅s^−1^), F is the Faraday constant (s⋅A⋅mol^−1^), T is the temperature (K), R is the gas constant (J⋅K^−1^⋅mol^−1^), α is the dimensional transfer coefficient with a value of 0.5 in this case, based on the assumption that the ratio of anodic to cathodic peak currents is 1, and ΔE is the peak-to-peak separation (mV). Conversely, the electron transfer rate constant, k_0_^′^, was determined using Equation (3), as described in previous studies [28,32]:

(3) $$k_{0}^{\prime }=\frac{RT}{n^{2}F^{2}ACR_{ct}}$$

where n is the number of electrons involved in the reaction, A is the electrode surface (cm^2^), C is the concentration of the redox couple (mol⋅cm^−3^), and Rct represents the resistance to electron transfer (Ω). Table 2 shows the voltammetric and impedimetric heterogeneous electron transfer rate constants (k_0_ and k_0_^′^, respectively) for the steps involved in the aptasensor assembly.

After proving the successful assembly of the aptasensor, its further use for PSA sensing based on sandwich-type strategy was also demonstrated using the same electrochemical techniques, CV and EIS (Figure 2C and Figure 2D, respectively). As shown in Figure 2C, the redox peak current decreases after incubation first with PSA and then, more abruptly, with Apt–HRP which indicates the decrease in the electron transfer because of the PSA and Apt–HRP presence. It was confirmed by the reduction in the electroactive surface area of this biosensor from 9.5 mm^2^ to 9.3 mm^2^ and 7.6 mm^2^ after sequential incubations with PSA and Apt–HRP, respectively. In addition, this behaviour correlates with EIS (Figure 2) illustrating how the diameter increases as recognition of the target analyte (932 Ω) and the labelling probe (1100 Ω) occurs (Figure 2(Db) and Figure 2(Dc), respectively).

**Figure 3:**
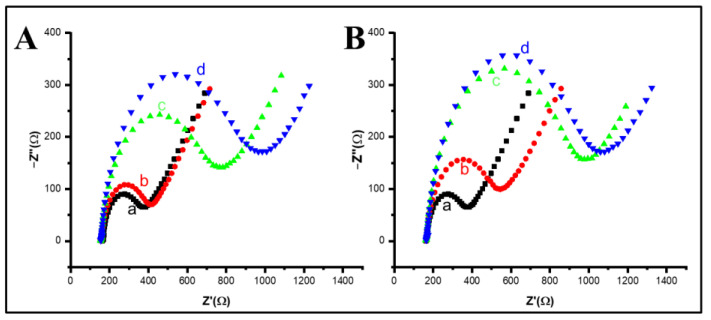
Nyquist plots of Apt–ADA/CD-AuNFs/rGO/SPCE electrode (**A**) before (a) and after sequential incubation with NaOH (b), MCH (c), and PSA (d). Nyquist plots of Apt–ADA/CD–AuNFs/rGO/SPCE electrode (**B**) before (a) and after sequential incubation with ADA-COOH in NaOH (b), MCH (c) and PSA (d). Measured in 0.100 M KCl solution containing 5 mM K_3_[Fe(CN)_6_]/K_4_[Fe(CN)_6_] (1:1).

**Figure 4:**
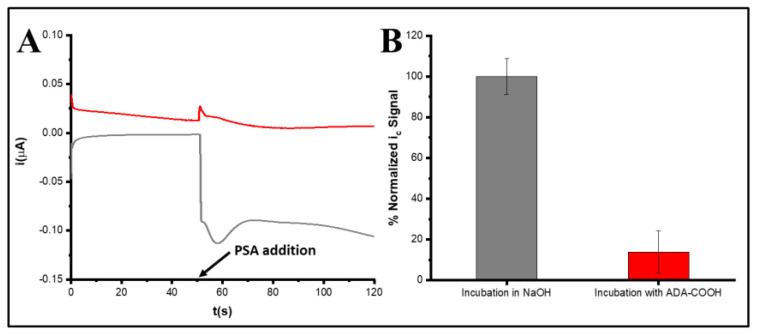
Amperometric responses (**A**) and relative amperometric analytical signal (**B**) of the aptasensor, previously incubated with NaOH (grey) or ADA-COOH in NaOH (red), towards PSA.

The supramolecular nature of the immobilization strategy used here to assemble the aptasensor was demonstrated, and several electrochemical experiments were performed; the results are shown in Figure 3 and Figure S3 (Supporting Information). To this end, the possible displacement of the aptamer already immobilized in the electrode through host–guest interactions was studied by using cyclic voltammetry (Figure S3A,B), electrochemical impedance spectroscopy (Figure 3A,B) and amperometry (Figure 4A). To do this, the sodium salt of 1-adamantanecarboxylic acid (ADA-COOH) was employed as a competitive guest molecule due to its high affinity for CDs. As is illustrated in Figure 3A, incubation of the Apt–ADA/CD–AuNFs/rGO/SPCE electrode in NaOH solution did not cause a significant change in the charge transfer resistance of this surface. On the contrary, incubation with ADA-COOH noticeably increased the charge transfer resistance (Figure 3B), suggesting that the Apt–ADA capture receptor was significantly replaced by the negative-charged adamantane derivative from their complex with CDs, causing a large electrostatic repulsion to the diffusion of the electrochemical probe to the sensing surface.

Such a replacement was also confirmed by EIS, by measuring the charge transfer resistance of the electrodes, previously incubated or not incubated with ADA-COOH, after affinity recognition of the PSA. As can be observed in Figure 3A, a significant increase was observed for the semicircle diameter in the Nyquist plot corresponding to the electrode after incubation with the PSA, suggesting a large recognition of the biomarker by the aptamer-enriched surface. However, a minor increase in the charge transfer resistance was observed for the ADA-COOH-treated electrode after incubation with the PSA (Figure 3B). This fact suggests a minor association of the biomarker to the electrode surface, probably caused by the low density of aptamer molecules on this sensing interface after replacement with the adamantane derivative. These hypotheses were also confirmed by CV measurements, as illustrated in Figure S3A,B in Supporting Information.

Amperometric measurements of the sandwich-type biosensing assembly were measured with Apt–ADA/CD–AuNFs/rGO/SPCE electrodes, previously incubated or not incubated with ADA-COOH. No significant amperometric signal was measured using the electrode previously treated with the adamantane derivative, suggesting that this electrode was not able to recognize PSA due to the absence of capture aptamers on their surface (Figure 4). However, a large amperometric signal was recorded with the electrode that was not incubated with ADA-COOH, demonstrating that the affinity receptor remains supramolecularly immobilized on this surface, and thus increased recognition of the biomarkers was achieved.

**Figure 5:**
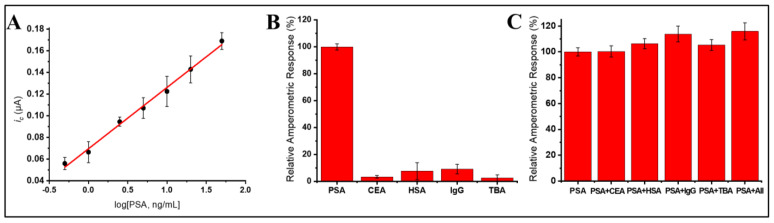
(**A**) Calibration plot for the aptasensor towards PSA in 0.1 M sodium phosphate buffer, pH 7.0. (**B**) Relative amperometric response of the aptasensor toward 50 ng·mL^−1^ PSA and 100 ng·mL^−1^ of CEA, HSA, IgG, and TBA, respectively. (**C**) Relative amperometric response of the aptasensor toward PSA and mixtures with other potential interfering proteins at the same concentration cited above.

The aptasensor with supramolecular architecture was then evaluated for the amperometric determination of PSA by using a sandwich-type scheme with the Apt–HRP conjugate as a signalling element. As Figure 5A illustrates, the cathodic amperometric responses proportionally intensified with the logarithm of the PSA concentration, according to the following equation:

i_c_ (μA) = 0.052·log[PSA, ng/mL] + 0.069 (R^2^ = 0.994, n = 10)

This aptasensor showed a linear range of response toward the logarithm of PSA concentration over the range of 0.5 to 50 mg/mL. The limit of detection (LOD) was calculated as 0.11 ng/mL using the IUPAC rules [33]. Upon comparison with the previous methods listed in Table 3, this newly developed aptasensor possesses an acceptable analytical performance, which is mainly attributed to a trade-off between obtaining more selective signals despite the decrease in sensitivity. The developed aptasensor is suitable for the detection of PSA in both serum and urine, where PSA concentrations range from 0.1 to a maximum of 10 ng/mL.

In order to demonstrate the suitable use of the proposed PSA aptasensor, its reproducibility, selectivity, and stability were studied. The aptasensor showed good reproducibility between days, with a relative standard deviation of 12.4% toward a 20 ng/mL PSA solution (n = 10, different electrodes). The good selectivity of the sensor was confirmed by the comparison of the relative amperometric response of 50 ng/mL PSA against 100 ng/mL of the potentially interfering substances found in human serum such as carcinoembryonic antigen (CEA), human serum albumin (HAS), immunoglobulin G (lgG), bovine serum albumin, and thrombin (TBA). As displayed in Figure 5B, the aptasensor barely showed a signal for any analyte other than PSA. In addition, the interferences on the PSA signal caused by the presence of interfering biomolecules were also studied, observing that the analytical response of PSA aptasensor was not statistically affected in most of the cases, with the exception of IgG (Figure 5C). Finally, the stability of the PSA aptasensors was probed through their storage in dry conditions at 4 °C. Figure S5 illustrates that the aptasensor can preserve its initial sensing capability after 35 days toward 20 ng/mL PSA.

## 3. Conclusions

Here, we reported an innovative sandwich-type electrochemical aptasensor based on supramolecular immobilization of affinity bioreceptors through host–guest interactions using adamantane-modified aptamers as capture elements and an aptamer-HRP conjugate for labelling. As a proof-of-concept, this strategy was successfully evaluated for the construction of an electrochemical aptasensor for PSA, utilizing one-use electrodes covered with a highly electrocatalytic GO/AuNF nanocomposite. This strategy opens new opportunities for the development of new aptasensors using synergistically adamantane-modified bioreceptor complexes, along with different labelling elements for the specific and sensitive determination of target analytes.

## 4. Reagents and Apparatus

Carbon screen-printed electrodes (SPCEs) and graphene oxide (GO) were purchased from Orion High Technologies. The prostate-specific antigen (PSA) was acquired from MERCK-Millipore. The specific anti-PSA aptamer modified with amino primary moieties at the 5′ end (5′-[AmC6]TTTTTAATTAAAGCTCGCCATCAAATAGCTTT) [45], N-hydroxysuccinimide (NHS), 1-ethyl-3-(3-dimethylaminopropyl)carbodiimide hydrochloride (EDC), 1-adamantanecarboxylic acid, 6-mercapto-1-hexanol (MCH), horseradish peroxidase (HRP), glacial acetic acid, sodium acetate, ethylene glycol, NaBH_3_CN, hydroquinone (HQ), H_2_O_2_, K_3_[Fe(CN)_6_], and K_4_[Fe(CN)_6_] were purchased from Sigma-Aldrich (St. Louis, MO, USA). NaHCO_3_, Na_2_CO_3_, MgCl_2_·6H_2_O, and m-NaIO_4_ were purchased from Panreac. Tetrachloroauric (III) acid hydrate (HAuCl_4_·XH_2_O) and heptakis(6-deoxy-6-thio)-β-cyclodextrin (CD) were acquired from Across Organic and Cyclolab, respectively. NH_4_Cl, NaH_2_PO_4_·2H_2_O, Na_2_HPO_4_, H_3_BO_3_, and KCl were acquired from Sharlau (Barcelona, Spain). Finally, NaOH and NaCl were purchased from Gerbu (Heidelberg, Germany).

Electrochemical measurements were performed with a PalmSens4 potentiostat using the software PSTrace 5.7 (PalmSens, Houten, The Netherlands). High-resolution field emission scanning electron microscopy (FE-SEM) was performed with a JEOL JSM 7600F microscope (JEOL, Tokyo, Japan).

- Preparation of the anti-PSA aptamer-adamantane conjugate (Apt-ADA).

To prepare the anti-PSA aptamer–adamantane conjugate (Apt-ADA), 1 mg NHS and 1 mg EDC were dispersed in 250 µL of 50 mM sodium phosphate buffer, pH = 6.0 and, afterwards, 25 µL of 24 mM 1-adamantanecarboxylic acid in 0.1 M NaOH were added. The mixture was stirred for 30 min and then 250 µL of 200 µM anti-PSA aptamer solution in the previous buffer was added. The mixture was stirred at room temperature for 1 h and then at 4 °C overnight. The resulting solution was dialyzed using a 3K Amicon Ultra-05 centrifugal filter (EMD Millipore Co., Madrid, Spain) in 1.4 M NaCl, 30 mM KCl, and 0.1 M sodium phosphate buffer, pH 6.0. Finally, to reach 100 µM as the final concentration, the solution was brought to a total volume of 500 µL. Before use, the Apt–ADA conjugate was folded to the hairpin structure as described [46,47].

- Preparation of the anti-PSA aptamer–horseradish peroxidase conjugate (Apt–HRP).

To prepare the anti-PSA aptamer–horseradish peroxidase conjugate (Apt–HRP), 2 mg HRP and 4 mg m-NaIO_4_ were dissolved in 20 mL of 50 mM acetate buffer at pH 5.0 and stirred in the dark for 1 h. Afterwards, 2 mL of ethylene glycol were added, and the mixture was stirred under the same previous conditions. The solution was then dialyzed using 50 mM borate buffer, pH 8.8, through a 10K Amicon Ultra-05 centrifugal filter to a final volume of 2 mL and concentration of 1 mg oxidized HRP/mL. Then, 100 µL of the anti-PSA aptamer previously folded to the hairpin structure [46,47] were mixed with 500 µL of 1.0 mg/mL oxidized HRP and stirred for 30 min in the dark at 4 °C. Next, 100 µL of 10 mM NaBH_3_CN was added and the solution was stirred, under the same conditions, for 4 h. The resulting solution was dialyzed using a 10K Amicon Ultra-05 centrifugal filter in a 5 mM MgCl_2_ 0.1 M sodium phosphate buffer at pH 7.5, reaching a final volume of 500 µL and concentration of 10 µM.

- Preparation of aptamer-functionalized electrodes (MCH/Apt–ADA/CD-AuNFs/rGO/SPCE).

First, the activation of the electrodes was carried out by chronoamperometry at a constant potential of 1.7 V for 60 s in Na_2_HPO_4_/NaH_2_PO_4_, pH 7.4, 0.1 M. After the chronoamperometry, the electrodes are rinsed with Milli-Q water and left to air dry. Previously activated SPCE were coated with reduced graphene oxide (rGO) through electrodeposition of GO by dropping 50 µL of 1.0 mg/mL GO dispersion in 0.1 M sodium carbonate buffer, pH 9.0 on the electrode surface and then applying 5 scans of cyclic voltammetry between −1.5 V and 0.6 V at 25 mV·s^−1^ [47]. Afterwards, the rGO/SPCE electrode was washed with MilliQ water and dried before sequentially dropping 60 µL of a freshly prepared aqueous solution of 0.1 M HAuCl_4_ in 1 M NH_4_Cl using amperometry at − 3 V for 20 s. The AuNFs/rGO/SPCE was then washed and dried.

In order to functionalize the AuNFs/rGO/SPCE electrode with the anti-PSA aptamer, 8 µL of 4 mM of thiolated β-cyclodextrin were incubated on the working electrode for 4 h at 4 °C in a humid chamber. After washing, 8 µL of 30 µM Apt–ADA complex was incubated on the working electrode surface at 4 °C in a humid chamber for 1 h. Finally, to avoid non-specific adsorption on the electrode surface, mercaptohexanol (MCH) was added as blocking agent. To this end, 8 µL of 1 mM MCH was dropped onto the working electrode surface and allowed to interact for 45 min at 4 °C in a humid chamber. Afterwards, the electrode was rinsed with MilliQ water and completely dried before use.

- Amperometric determination.

The PSA standard solutions were prepared in 0.1 M sodium phosphate buffer, pH 7.0. Then, 8 µL of each PSA standard solution was dropped on the Apt–ADA/CD–AuNFs/GO/SPE working surface and kept at 4 °C in a humid chamber for 1 h. The electrode was then washed with the buffer and dried before 8.0 µL of 5 µM anti-PSA Apt–HRP conjugate solution was added. The electrode was incubated for 30 min at 4 °C in a humid chamber. After incubation, the electrode was washed and dried.

To drive analytical measurements, 45 µL of 1 mM hydroquinone in 0.1 M sodium phosphate buffer, pH 7.4, were added to the electrochemical cell and amperometric signals were recorded at −0.2 mV after the addition of 5 µL of a freshly prepared 50 mM H_2_O_2_ solution.

The difference between the stabilized signal and the signal after H_2_O_2_ addition is the intensity of the amperometric signals. The relative amperometric signal, which consider the non-specific signal, has been calculated as follows:

$$Relative\;Amperometric\;Response\;\%=\frac{\left(i_{specific\;signal}-i_{non-specific\;sigal}\right)}{i_{specific\;signal}}\times 100$$

The results obtained from the selectivity and interference assays were statistically compared using variance (F) and mean analysis (*t*-student).

## Figures and Tables

**Table 1:** Optimal values for the preparation of the PSA aptasensor.

Optimized Parameter	Range	Optimal Value
[Apt–ADA]	5–40 µM	30 µM
Incubation time of Apt–ADA	15–90 min	60 min
Incubation time of PSA	15–90 min	60 min
[Apt–HRP]	0–15 µM	5 µM
Incubation time of Apt–HRP	15–60 min	30 min

**Table 2:** Parameters estimated for SPCE and EIS in 5 mM [Fe(CN)_6_]^4−/3−^ in KCl during the aptasensor fabrication.

	ΔE (mV)	Ipa/Ipc	K_0_ 10^−3^ (cm/s)	R_ct_ (Ω)	A (cm^2^)	K_0_^′^ 10^−3^ (cm/s)
Bare SPCE	201	0.96	0.74	498	0.122	0.88
rGO/SPCE	176	0.92	1.00	471	0.138	0.82
AuNF/rGO/SPCE	121	0.93	2.41	25.6	0.177	11.74
CD-AuNF/rGO/SPCE	216	0.95	0.62	373	0.118	1.21
Apt-ADA/CD-AuNF/rGO/SPCE	297	0.83	0.30	681	0.078	1.00
MCH/Apt-ADA/CD-AuNF/rGO/SPCE	297	0.95	0.30	798	0.095	0.70
PSA/MCH/Apt-ADA/CD-AuNF/rGO/SPCE	302	0.94	0.29	932	0.093	0.61
Apt-HRP/PSA/MCH/Apt-ADA/CD-AuNF/rGO/SPCE	398	0.89	0.15	1100	0.076	0.64

**Table 3:** Comparison with previous methods for the electrochemical detection of PSA.

Detection System	Method	Linearity Range (ng/mL)	LOD (ng/mL)	Refs.
Apt-PSA/GQDs-AuNRs/SPCE	DPV	0.14–11.6	0.14	[34]
Anti-PSA/GQDs-AuNRs/SPCE			0.42	
Apt-PSA/MB/cDNA/GO-COOH/SPCE	DPV	0.001–100	6.4 × 10^−5^	[35]
Apt-PSA/MWCNTs-AuNP/SPCE	DPV	1–100	0.001	[36]
MIP-Ppy/AuE	DPV	0.01–4	0.002	[37]
Apt-PSA/AuNPs/THI/rGO/GCE	DPV	0.05–200	0.01	[38]
Ab_1_/CHIT-MOF/GCE and Ab_2_-QDs	DPV	0.001–100	4.5 × 10^−4^	[39]
BSA/Ab-PSA/NiFe PBA/AuNPs/GCE	DPV	0.0005–1	0.00023	[40]
MB/Apt-PSA (sterm-loop)/AuE	SWV	0.01–500	1.24 × 10^−3^	[41]
Apt-PSA/GA/AuNPs/Nafion/SPCE	EIS	0.05–50	0.0306	[42]
ITO/CTES/Apt-PSA	EIS	0.000001–0.0015	8.76 × 10^−6^	[43]
Ab_1_-Biot/Strep/Ppy/ITO and HRP/Ab_2_-PpyNPs	Potentiometric	0.001–40	7 × 10^−4^	[44]
Apt-ADA/CD/AuNFs/rGO/SPCE and HRP-Apt	Amperometric	0.5–50	0.11	This Work

Apt–PSA: PSA aptamer, Anti-PSA: PSA Antibody, GQDs: Graphene Quantum Dots, AuNRs: Gold Nanorods, SPCE: Carbon screen-printed electrode, MB: Methylene blue, cDNA: Complementary DNA, GO: Graphene oxide, MWCNTs: Multiwalled carbon nanotubes, AuNP: Gold nanoparticles, MIP: Molecularly imprinted polymers, Ppy: Polypyrrole, AuE: Gold electrode, GA: Graphene Aerogel, CTES: Carboxyethylsilanethiol, rGO; Reduced graphene oxide, CHIT: Chitosan, MB: Methylene Blue, MOF: Metallic organic framework, GCE: Glassy carbon electrode, Ab: Antibody, Biot: Biotin, Strep: Streptavidin, ITO: Indium tin oxide electrode, HRP: Horse radish peroxidase, ADA: Adamantane, CD: Cyclodextrin, AuNFs: Gold nanoferns.

## Data Availability

Data are contained within the article and Supplementary Materials.

## Supplementary Materials

The following supporting information can be downloaded at: https://www.mdpi.com/article/10.3390/molecules29194714/s1, Figure S1: SEM images of activated bare SPCE. Figure S2: Optimization of parameters for the electrode assembly and relative amperometric response for the aptasensor. Figure S3: Study of the supramolecular interaction between the CD-ADA complex in free adamantane excess. Figure S4. Fitting of the Nicholson and Perone data in ΔE values of a quasi-irreversible system. Figure S5: Effect of time of storage on the relative amperometric response of the aptasensor toward PSA.
