# Supplementary material for: Sandwich-Type Electrochemical Aptasensor with Supramolecular Architecture for Prostate-Specific Antigen
Source: Molecules. 2024 Oct 5;29(19):4714. doi: 10.3390/molecules29194714 (PMC11478330; doi:10.3390/molecules29194714)
Supplement: Supplementary file 1 [file molecules-29-04714-s001.zip › molecules-3158779-supplementary.pdf]

Supporting Information

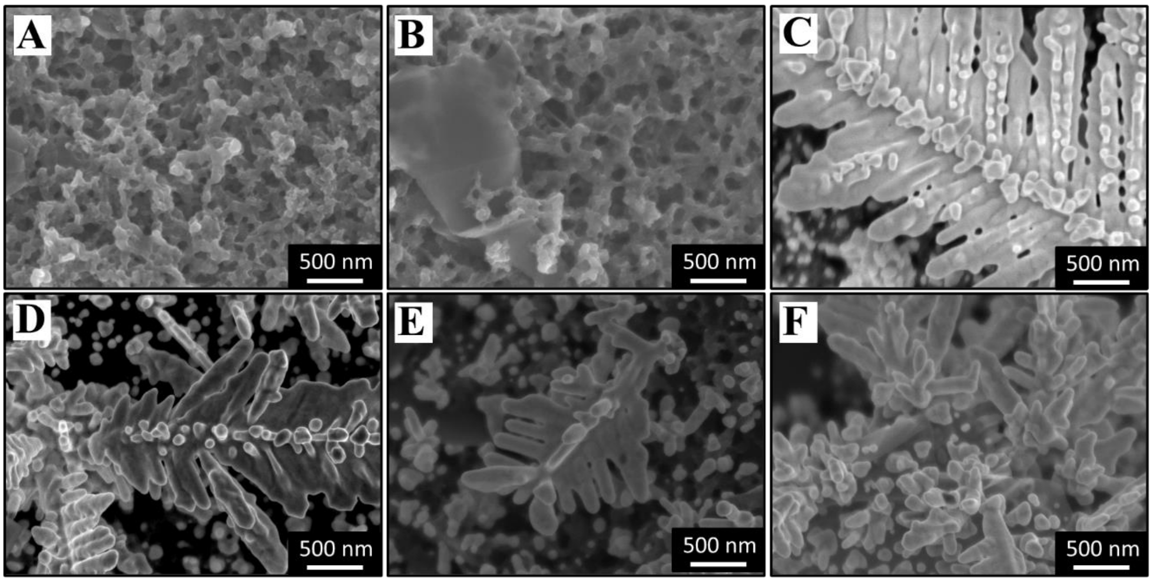

Figure S1. Representative FE-SEM images of bare CSPE (A), rGO/CSPE (B), AuNFs/rGO/CSPE (C), CD-AuNFs/rGO/CSPE (D), Apt-ADA/CD-AuNFs/rGO/CSPE (E), MCH/ Apt-ADA/CD-AuNFs/rGO/CSPE (F) at high magnification.

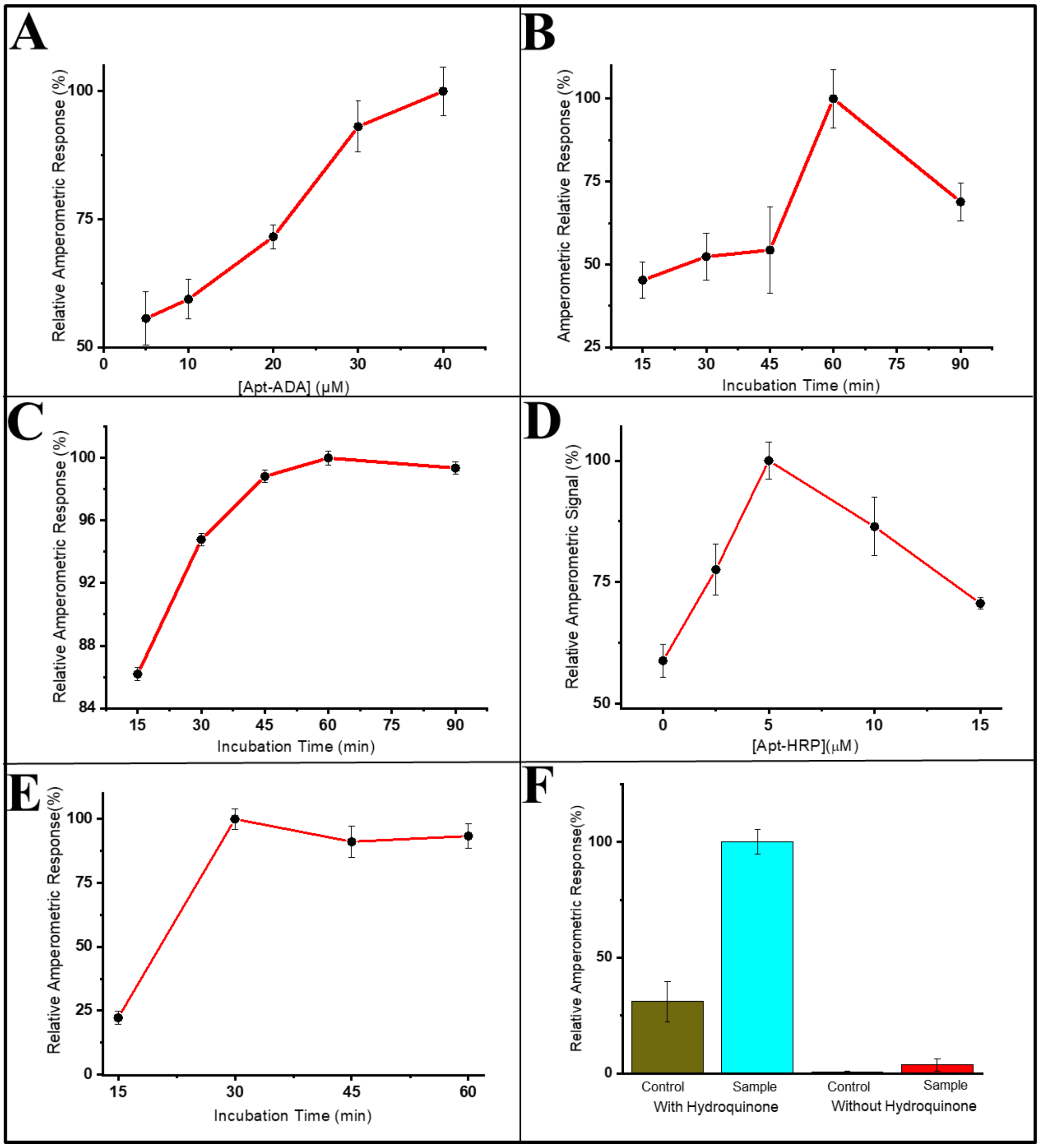

Figure S2. Optimization of Apt-ADA concentration (A), Apt-ADA incubation time (B), PSA incubation time (C), Apt-HRP concentration (D), Apt-HRP incubation time (E), and relative amperometric response for the aptasensor after sequential incubation with 20 ng/mL PSA or buffer (control), and with or without hydroquinone (F).

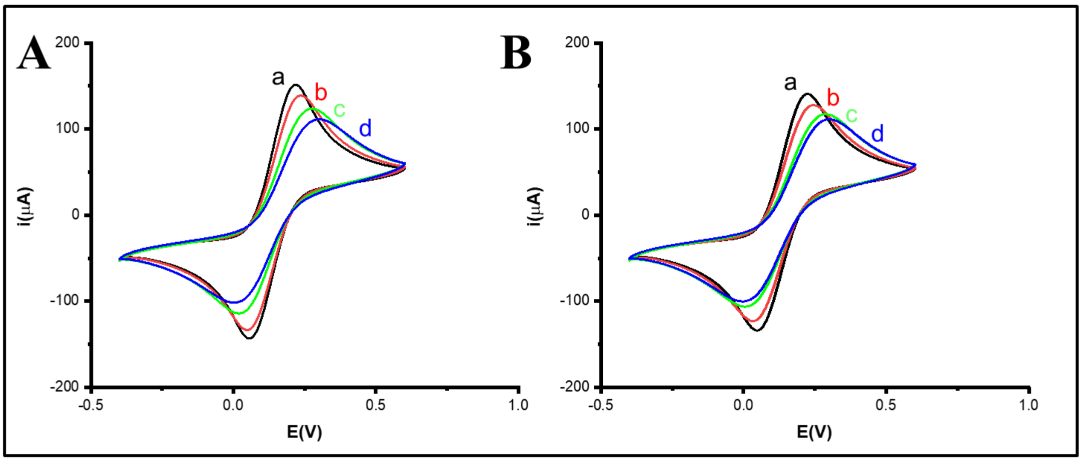

Figure S3. (A) Cyclic voltammograms of Apt-ADA/CD-AuNFs/rGO/CSPE electrode before (a) and after sequential incubation with NaOH (b), MCH (c) and PSA (d). (B) Cyclic voltammograms of Apt-ADA/CD-AuNFs/rGO/CSPE electrode after sequential incubation with ADA-COOH in NaOH (b), MCH (c) and PSA (d). Measured in 0.100 M KCl solution containing 5 mM $K_3[Fe(CN)_6]/K_4[Fe(CN)_6]$ (1:1) at scan rate of 50 $mV{\cdot}s^{-1}$.

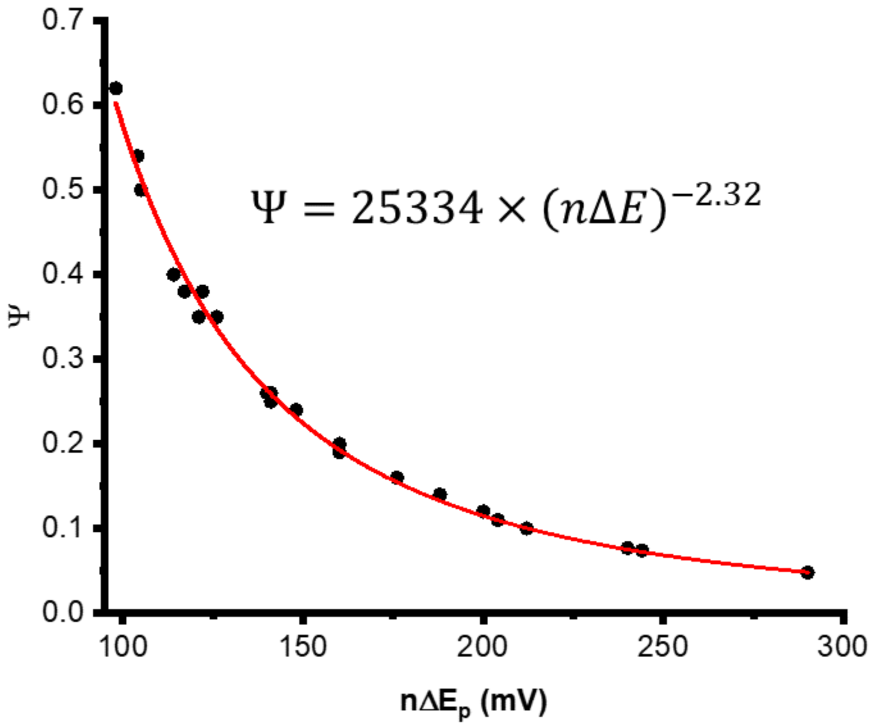

Figure S4. Fitting of the Nicholson and Perone data: Ψ vs. ΔE values.

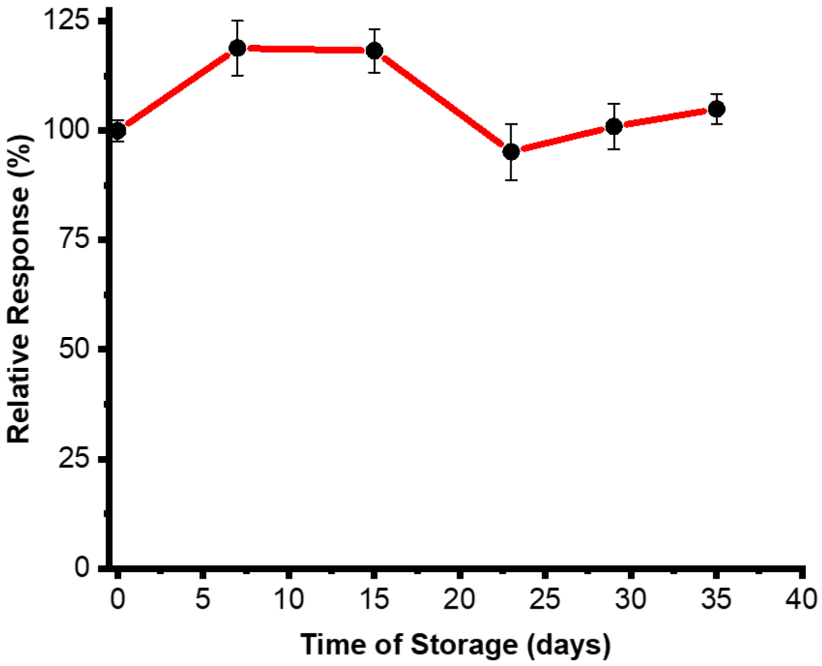

Figure S5. Effect of time of storage in dry conditions at 4ºC on the relative amperometric response of the aptasensor toward 20 ng·mL^{-1} PSA.
